# Patient data and patient rights: Swiss healthcare stakeholders’ ethical awareness regarding large patient data sets – a qualitative study

**DOI:** 10.1186/s12910-018-0261-x

**Published:** 2018-03-07

**Authors:** Corine Mouton Dorey, Holger Baumann, Nikola Biller-Andorno

**Affiliations:** 1 Institute of Biomedical Ethics and Medical History (IBME), University of Zurich, Winterthurerstrasse 30, CH-8006 Zurich, Switzerland; 2 Philosophy Seminar, University of Zurich, Zollikerstrasse 117, Zürich, CH-8008 Switzerland

**Keywords:** Agency, Ethics, Healthcare stakeholders, Health data, Justice, Patient rights, Reciprocity, Clinical registries

## Abstract

**Background:**

There is a growing interest in aggregating more biomedical and patient data into large health data sets for research and public benefits. However, collecting and processing patient data raises new ethical issues regarding patient’s rights, social justice and trust in public institutions. The aim of this empirical study is to gain an in-depth understanding of the awareness of possible ethical risks and corresponding obligations among those who are involved in projects using patient data, i.e. healthcare professionals, regulators and policy makers.

**Methods:**

We used a qualitative design to examine Swiss healthcare stakeholders’ experiences and perceptions of ethical challenges with regard to patient data in real-life settings where clinical registries are sponsored, created and/or used. A semi-structured interview was carried out with 22 participants (11 physicians, 7 policy-makers, 4 ethical committee members) between July 2014 and January 2015. The interviews were audio-recorded, transcribed, coded and analysed using a thematic method derived from Grounded Theory.

**Results:**

All interviewees were concerned as a matter of priority with the needs of legal and operating norms for the collection and use of data, whereas less interest was shown in issues regarding patient agency, the need for reciprocity, and shared governance in the management and use of clinical registries’ patient data. This observed asymmetry highlights a possible tension between public and research interests on the one hand, and the recognition of patients’ rights and citizens’ involvement on the other.

**Conclusions:**

The advocation of further health-related data sharing on the grounds of research and public interest, without due regard for the perspective of patients and donors, could run the risk of fostering distrust towards healthcare data collections. Ultimately, this could diminish the expected social benefits. However, rather than setting patient rights against public interest, new ethical approaches could strengthen both concurrently. On a normative level, this study thus provides material from which to develop further ethical reflection towards a more cooperative approach involving patients and citizens in the governance of their health-related big data.

**Electronic supplementary material:**

The online version of this article (10.1186/s12910-018-0261-x) contains supplementary material, which is available to authorized users.

## Background

Patients’ healthcare data is used by a range of stakeholders, for a variety of different purposes, and this picture is rapidly changing. Greater digital integration of large health datasets is advocated for its benefits to clinical research and healthcare practice, blurring the distinction between research activities and medical care [1]. The expected social benefits are estimated to be considerable, especially with genomics and precision medicine aiming at more targeted and safer treatment for patients. Consequently, new approaches to informed consent are being examined, to facilitate the collection and use of routine patient data into big health data networks. Indeed, it is unrealistic to obtain informed consent for secondary uses of patient data, when the purposes of such uses are not known at the time of data collection [2]. As a result, patients’ rights to be informed and to give consent before their data is shared may not be respected, infringing upon the fundamental human right to privacy. However, patients also have increasing access to medical information, and could thus take on a more active role regarding their health-related data, based on their patient rights not only to privacy, but also to agency and participation.

As the healthcare system relies increasingly on digital solutions [3], the rapid development of large patient data sets could have serious repercussions for individual patient rights, social group protection, and trust in physicians and public institutions [4]. It seems that many patients are unaware of possible conflicts of interest regarding data sharing, including for commercial purposes, and existing protective laws and ethical arguments do not fully address these new challenges [5]. New approaches to the ethical governance of patient data need to be envisioned and discussed. A first step would be to better understand the current ethical awareness of healthcare stakeholders (HCS) who contribute to the establishment of large patient data sets. Therefore, we have chosen to investigate empirically HCSs’ experiences and ethical consciousness, with regard to patient data, in the setting of clinical registries in Switzerland.

Clinical registries (CRG) are a good proxy for large patient data sets. They use observational methods to gather patient data in order to assess medical outcomes and processes at population levels [6]. They cover a large healthcare domain, extending from clinical quality improvement, safety monitoring and cohort studies, to clinical research and policy evaluation, and they are confronted with similar digital changes and challenges as those of the wider field of patient healthcare data. There is growing concern for patient rights, as it could be possible to re-identify specific individuals, when CRGs built with de-identified or anonymised data are linked, or include genetic information. There is also concern that aggregated information could stigmatize and harm some groups of patients and citizens, because of their disease, lifestyle or extra healthcare costs. HCSs should therefore be aware of their moral obligations when they decide to create or contribute to a clinical registry.

Switzerland is a country with robust privacy rights written in its Constitution “Everyone has the right to be protected against the misuse of their personal data” [7]. Healthcare data are considered as sensitive personal data. To collect and use them, it is necessary either to have a legal basis, to demonstrate a dominating public interest, to have informed consent, or to have anonymous or coded data [8]. In comparison to other countries such as the United Kingdom, Australia, Sweden or Denmark, CRGs are not extensively developed in Switzerland [9]. However, this is changing. The Health2020 report gives an overview of health-policy priorities in Switzerland for the next 8 years. It identifies four interdependent priorities to be developed incrementally: Quality of life, equality of opportunity, quality of care provision, and transparency. In short, better data is required to transform the healthcare system into one with increased efficiency and improved quality, whilst containing growing costs [10]. Therefore, initiatives in favour of e-health, database linkage and national data sharing are facilitating the collection and use of routine patient data, and consequently the development of CRGs. However, to date, there is no empirical data on HCSs’ real-life experiences of CRGs.

This paper reports a qualitative study designed to investigate HCSs’ ethical awareness regarding the management of patient data in Swiss real-life settings where CRGs are decided, created, managed and used. Whilst the literature reports an increasing involvement of patients with their personal data, the Swiss respondents did not seem to consider patient rights evolving into self-legislation and participation. Rather, scientific and legal matters seem to be their primary concern, regarding the creation and use of large patient data sets. This finding acknowledges the emergence of a tension between public biomedical research interests and patient rights regarding the newly emerging production and use of patient data. The study thereby motivates further normative reflections on the ethical approach that is taken for building large patient data sets, i.e. an approach that emphasizes a fair distribution of benefits and burdens amongst all stakeholders, patients and citizens included.

## Methods

A thematic analysis, derived from Grounded Theory, and using semi-structured interviews, was selected to explore HCSs’ individual experience and reflections [11]. The cantonal research ethics committee declared the study to bear no ethical risk (KEK-StV-Nr. 42/14). Participant information sheets were sent in advance by email to all possible interviewees. At the beginning of the interviews, consent forms and confidentiality agreements were explained, signed and exchanged.

In order to have a wide diversity of roles and experiences, a purposive sampling frame selected three targeted groups of HCSs:

- Group (M) comprises physicians involved in “Making” CRGs. Including two sub-groups, frontline physicians collecting data (sub-group M’) and data centre managers (sub-group M”);
- Group (R) includes people “Reviewing” CRG protocols in research ethical committees;
- Group (A) includes people “Asking” for CRGs i.e. sponsors, regulators and policy-makers who require, fund or control the creation of CRGs.

The study sample did not include CRG patients, as their identities were anonymous or coded, i.e. not accessible. Thus, the abbreviation HCS used for respondents does not include patients. Recruitment was based on the information provided by the Swiss Medical Association “FMH” platform for CRGs [12], through the first author’s direct contacts and by snowballing. Sample size was determined by data saturation, i.e. the point at which additional data fails to generate new information. A range of 15 to 25 interviews was foreseen, with group M expected to be the largest group, as its members are the closest to patients.

Documents to participants were produced in 3 languages: French, English and German. The participant information sheet included a definition of clinical registries based on the U.S. Agency for Healthcare Research and Quality (AHRQ) document. At the time of the interview, this definition was read and the interviewees were asked to fill in a matrix, based on the AHRQ definition, to identify their own CRG experience. An additional file shows this in more detail [see Additional file 1].

The interview topic guide was developed with the help of an ad-hoc literature review identifying an initial framework of possible ethical issues to be raised by CRG stakeholders. An additional file shows this in more detail [see Additional file 2]. The topic guide included three items:

- Participants’ personal experience of CRG. Assessed with open-ended questions;
- General CRG issues. Interviewees were encouraged to think aloud. Prompt cards were used to highlight potential ethical issues and blank cards were used to record other emerging issues;
- Possible recommendations for future CRGs.

The topic guide was reviewed by external experts in qualitative research and epidemiology, and tested with native-speakers of the three languages. Prompt cards were used to facilitate discussion and communication with stakeholders who may not be at ease expressing themselves within the lexical field pertaining to ethics. Where possible, interviewees were asked to sort these cards by relevance to further assess their attitudes and beliefs. An additional file shows an example of the topic guide used for group M [see Additional file 3]. For groups R and A, the first item was slightly modified, so as to be more appropriate to these participants’ roles and experiences. To ensure a relaxed and trusting atmosphere, a methodology of face-to-face interviews, at the interviewees’ location, was chosen. Interview proceedings were recorded in a qualitative research journal.

Interviews were conducted by one of the authors (CMD), who is trained in qualitative methods. Interviews took place between July 2014 and January 2015, and were conducted in English, French and German. Thirty candidates were contacted, 22 accepted to participate. Reasons provided by those who declined participation, included lack of time (*n* = 5) and a lack of experience with clinical registries (*n* = 3 who then provided the name of a more appropriate candidate). Saturation was recognised after the first 15 interviews, however the study continued as 4 more interviews had already been planned. To confirm saturation, three more interviewees, from fields outside of the initial sampling frame (quality management, patient association, clinical ethicist) were selected using a discriminative sampling approach, and interviewed. The interviews lasted on average 59 min (median 60 min). All interviews were audio-recorded and transcribed verbatim. The transcription was done on a continuous basis and data were exported into NVivo™ software for Mac. Coding and categorisation processes were gradually updated. First-level codes were regrouped in an iterative process between data collection and analysis. Memos were written throughout the research process.

The first six interviews were used for developing the coding book. Additional coding was added when necessary with later interviews, and previous interviews were reviewed accordingly to ensure consistent coding for all transcriptions. Facilitated by NVivo™, the thematic analysis procedure used successive matrices to cross-tabulate different categories of response. Our interpretation followed a mix of deductive (initial framework-informed) and inductive (theory-generating) approaches, with a continuous comparison method to interpret expected and emergent themes [13]. The final analysis followed the OSOP method [14], resulting in a map of key themes. This contained explanations of patterns and linkages, analysis of deviant cases, and allowed us to generate inputs for an emergent theory.

The first author coded all the transcripts, developed themes and proposed the final analysis. To finalize the coding book, colleagues, acknowledged at the end of the article, independently coded a sample of de-identified transcripts in English, French and German. As educational background can influence qualitative interpretation, it is important to note that the first author has a medical background and further education in bioethics and empirical research. The co-authors, who enriched and validated the analysis, have backgrounds in biomedical ethics, philosophy and medicine.

## Results

### Respondent characteristics

Table 1 shows the participants’ demographic information. Health domains were diversified. The region of Zurich and the public health sector were the most represented. A majority of interviewees were male and had a medical background. Most of the interviewees had experience in more than one type of CRG. A few interviewees (mainly sub-group M’) did not find it relevant to differentiate between CRGs for research and CRGs for quality improvement, as for them, these different goals require the same data, and indeed, the patients involved are the same.

**Table 1:** Characteristics of the interviewees (*n* = 22)

Male (n, %)	19 (86%)
Age (median, range)	55 (39–68)
Years of experience with clinical registries (median, range)	14 (1.5–27)
Experience working abroad > 1 year (n, %)	12 (55%)
Number of registries currently involved in (median, range)	2 (1–6)
Current main role regarding CRG (n)	
First line data collectors (M’)	6
CRG data center manager (M”)	5
Initiators/ sponsors (politics, federal administration, patient organisation, quality management) (A)	7
Reviewers (cantonal ethics committee, clinical ethicist) (R)	4
Education background (n)^a^	
Medical doctor	16
PhD science	5
Economy	1
Law & humanities	2
Nurse	1
Health care (HC) domain (n)	
Main Medical fields	
Anaesthetics	1
Cardiology	1
Dermatology	1
General practice	1
Infectious diseases	1
Nephrology	1
Paediatrics	1
Public Health	2
Other HC fields	
Data management direction	2
Quality management	1
Health administration	3
Ethics	4
Health policy	3
First language (n)^a^	
German	17
French	6
Italian	2
Places of work (n)	
Geneva	1
Lausanne	3
Fribourg	2
Bern	4
Zürich	12
Sector (n)	
Public	19
Private	3

^a^Interviewees could satisfy 2 characteristics. CRG: clinical registries

### Categories and gradual findings

Five anticipated categories, identified in the initial framework of expected ethical issues, where used as prompt cards:

- patient information
- data ownership
- trust
- moral obligations
- confidentiality

In the first interviews, additional issues arose concerning data set linkages, data interoperability, data sharing, communication between stakeholders, feedback of results, quality of data, utility of CRG, funding issues and legal constraints. In order to take into account these issues and to fully explore their ethical dimensions, two supplementary prompt cards were added after the first 6 interviews:

- communication-networking
- long-term value

Categorising was based on these seven prompt cards, and completed by the following emergent categories: perceived CRG definition and knowledge, legal aspects, transparency, governance, beneficence, similarities with biobank, empathy with patients, and a parking-lot category named “other possible ethical issues”, which included elements of card strategy and prioritization.

### Thematic analysis

The data was very rich. Comparing and looking for interactions across categories in the light of the research question on HCSs’ ethical awareness, the analysis identified three broader themes: Respondent behaviour, attitude and strategy. *Behaviour* described how HCSs worked or could act in the context of the production and use of the CRGs. The theme *attitude* revealed HCSs’ thoughts, beliefs and values towards themselves, peers, patients, and society, i.e. disclosed something about their moral obligations. *Strategy* exposed respondents’ reactions when confronted with a tension between their actions and their beliefs.

#### Respondent behaviour

All interviewees emphasized the need to intervene in CRG management with strong legal and operational rules. Legal concerns were extensively discussed, despite the absence of specifically legally oriented questions in the topic guide. (Table 2) Swiss law confers upon HCSs the right to record and use CRG data. It assures that HCSs respect privacy rights, confidentiality, anonymity-coding rules and informed consent. In effect, this judicial backbone strengthens professional deontological rules requiring that HCSs first do no harm and abide by their professional secrecy duty.

**Table 2:** Healthcare professionals’ behaviour – Legal norms

Necessity of legal norms:	Interviewee comments (A, M’, M”, R indicate group affiliation)
For opt-out preferential option	[Sub-group M”]. *“First of all we had to fight for the legal fundaments, because if you don’t have a legal fundament then in cantons some hospitals would simply refuse to deliver the data, which would then lower our response rate below the international acceptable limit. So, legal, a legal baseline is very important…There is a moral obligation to use data, to improve quality of life of patients, therefore to have good quality data and completeness. It means obligation to have an opt-out...There is a conflict of moral obligation with the data protection officer who wants first to protect individual privacy. That is why a law will help for [name of the clinical registry] registry.”*
As a basis for confidentiality	[Sub-group M’]: *“Confidentiality is very important to get that your patient information is given and that the patient is not disappointed. Depends on what you do, I mean, organ transplant recipients everybody knows they are transplanted so there is not that much to hide. With HIV that’s much different. You have more concern in the HIV cohort study.”*
To better define research	[R] [translation] *“There is a clear contradiction in the definition of a clinical trial in the law and in the ruling order. As a result, researchers want to take advantage of this…we have recurring discussions on this question, whether it is research or not. And researchers put a lot of energy and intellectual efforts to argue that in this particular concrete case, it is not research.”*
With guidance for implementation	[Sub-group M”] *“Well I mean, there is the legislation and so on, but maybe there is probably not enough guidance in practice, that is known by the people who are developing registries and using them.”*
Applied with an idea of prudence	[Sub-group M’] “*That’s an ongoing discussion. Because it’s a pain. … if you have a question that is beyond an individual patient’s treatment, basically it’s science. … So my interpretation is that we have to ask for every project for specific approval for the specific question, which is what I am doing...but not everybody in the cohort study is of my opinion and we have heated discussions because it makes a big difference if you have 40 projects running in this cohort study which are scientifically looking at the cohort study and all these 40 need ethical approval or don’t. … you can go through any kind of audit and I know if you don’t have ethical permits you are lost. You are dead before the game even starts; because for a lawyer, if you have no document, you’re dead.”*

Depending on the type of CRG as identified in the Additional file 1 [see Additional file 1], legal requirements are different regarding data protection, confidentiality, patient consent and ethical review. When CRGs were legally mandatory for healthcare statistics, the respondents considered them as good examples of CRGs with an underlying stable structure and stable financial basis. Additional ad-hoc research purposes could thus be managed by simply adding other predefined items into the case report forms of the initial registry. Regarding non-mandatory CRGs, the interviewees were fighting for a legal basis for opt-out consent procedures, rather than an insistence upon opt-in consent, to facilitate CRG recruitment. Some HCSs mentioned their discretionary power in addressing confidentiality or informed consent issues depending on the types of patients included. For instance, the HIV registry was managed with a high level of confidentiality due to the risk of discrimination, whereas in the mandatory Swiss Transplant Cohort patients’ confidentiality was not a major issue due to the transparent transplantation context.

Major concerns pertained to the difficult distinction between clinical care and research CRGs. According to current regulation, only research CRGs need to be reviewed by the corresponding Regional Research Ethical Committee. In practice, the question raised by respondents was whether they should submit certain CRG projects to the research ethical committee. Indeed, their classification as research projects depends on the interpretation of the Swiss Human Research Act (HRA), as illustrated in the following examples:

- research CRGs with anonymous or coded data,
- quality CRGs delivering generalizable output,
- CRGs including “de-identified” biomarkers and genomics data,
- and all secondary uses of CRG collected data for research projects that were still unknown at the time of patient recruitment.

Confronted with this issue, HCSs behaved differently. Many viewed the obligation for a written informed consent, as too burdensome. Group M defined research based on the purpose and use of the CRG. In contrast, participants from groups A and R preferred to classify CRGs with non-identifiable data as monitoring tools, and not as research, stating that they could subsequently correct their approach with a retrospective ethical authorization if CRG results were published as research.

Besides these legal aspects, interviewees’ behaviour focused on the operational management of the CRGs, i.e. data quality, standardization and completeness, system interoperability, and financial conditions. (Table 3) Interviewees pointed out the risks of collecting the wrong data, or in the wrong way and generating waste (“data cemeteries”). The prospect of not using CRG information was considered as bad as misusing it. The further that interviewees were from the data collection process, the stronger was their doubt about CRG quality and value. All interviewees insisted on the necessity of working with a steering committee that would set the rules of good clinical practice, data access and research authorship.

**Table 3:** Healthcare professionals’ behaviour – Operational rules

Good operational management	Interviewee comments (A, M’, M”, R indicate group affiliation)
Importance of data quality	[Sub-group M’, *principal investigator]: “The crucial aspect of a registry is what kind of data do you put into this registry, and how well is this data controlled, and how good is the quality of this data in the end. This is what really counts… It is difficult you know; I’m continuously involved if there are some questions about definitions. Definitions always evolve. How do you, what kind of data point do you collect?”*
Trust in quality	[A]: *“My special problems with the evaluation registries as we call them, is that the physicians deliver the data to these registries, enough data, good quality data and that you have registries that you can use! That was always a problem and it is a problem: how to make them mandatory or how you can guarantee that the data are full and good. That is always the problem.”*
Good management needs human and financial resources	[R]: [translation] *“Money is necessary, for infrastructure and people, but public services are always reluctant. …With the National Research Fund, it is discouraging, they don’t want to engage themselves in the long term…a better coordination should exist between institutions and the National Research Fund, with a guarantee at the launch of a project that institutions will take over later.”*
Issue of definition of quality	[Sub-group M’]: [translation] *“It is difficult to measure quality in medicine. What is it? Is it patient satisfaction? Is it cost-effectiveness? Because when parliamentarians speak about quality, it is completely wrong: for them, it is quality – price ratio; when they think quality, it is profitability, and for me it is not. Quality has nothing to do with money…because if you want true quality, it would be expensive.”*
Steering committee to set the rules	[Sub-group M’]: *“You need some clear rules how will these data and samples be used, you know, by whom? And we have actually modelled ourselves a bit along the HIV cohort study which has a scientific committee; so, whenever somebody has a research question, he has to go there, has to write the proposal, we review the proposal and we accept the proposal or not.”*
Utility is essential	[Sub-group M”]: *“If we collect data, we have to organize everything that we can create as much information out of this data as possible. So, I think it is, it’s only serious to collect data if they can be used for something. If they are just collected and if they are not, cannot contribute to improve the system, then it is, I think it is not ethical to collect them.”*

#### Respondent attitude

A majority of interviewees declined to comment on the card on *moral obligations*, touching and looking at the card, but then moving on to comment on another card instead. Some HCSs considered the word “moral” too judgemental or inappropriate, and refused the “outing” on moral obligations. Nevertheless, morally connoted words like “right” and “wrong” were frequently used when they thought aloud about patient information, confidentiality, trust and long-term value. A few HCSs referred to moral obligations as the roof governing the whole CRG activity. Interviewees’ attitude varied between the different groups, based on their proximity to patients and the existing deontological code for physicians.

i. Attitude towards themselves and other HCSs (Table 4)

**Table 4:** Healthcare professionals’ attitude towards themselves and peers

Attitudes	Interviewee comments (A, M’, M”, R indicate group affiliation)
Moral obligations inherent to political engagement	[A] [translation] *“They [mandatory hospital CRGs] are mandatory statistics, therefore it seems relatively obvious as moral obligations to maintain them.”*
	[A] *“Our role is to propose to the parliament a law, that is useful, the most useful possible [for CRG]. That’s I think our most noble and our most important duty.”*
Necessity to better inform patients	[Sub-group M”]: *“I think the patients are not well informed and I think maybe there are some fields, which could be destroyed if there would not be an objective information. You know, at the moment, there are a lot of news for example showing the data or pictures from persons are provided on the internet because they have been taken out of clouds or whatever. And I think this increases the fear, and I think it will be very important to inform patients on what data are stored, why they are stored and that they cannot be identified for example.”*
Norms can be burdensome	[Sub-group M’]: *“We don’t need new regulation because as a doctor, as a lawyer, you have your professional obligations to keep your clients or patients’ data secret. So, if you don’t do that, you can be brought to court nowadays, so I don’t see what it changes if the patients must sign 5 such forms entering a hospital, on biobank, on whatever registry... it is counterproductive. You want to have an informed and empowered patient but it’s completely the other effect, you induce with paperwork. Nobody can understand the legislation.”*
Issue of transparency and communication	[Sub-group M”] *“I am absolutely convinced that physicians do not want to have this level of transparency, because everyone in this country who is allowed by the patient to load, enter to his file, can see what the other physician did, and “untransparency” is a very important thing in the health care system.”*

Group A said that moral obligations were implicitly handled in their political engagement when they were making laws. They recognized the moral obligation to apply legal norms, and to modify the legislation if necessary. Group M was aware of professional duties to inform patients, to respect secrecy, to accept peers’ scrutiny and finally to participate in CRGs as in other types of activities that would improve healthcare quality for patients. With regard to patient information, their motivation was not only deontological, but also utilitarian to maintain a trustful relationship for increasing patient participation in the registry and maintaining patients in a CRG cohort. A minority of interviewees considered that the addition of federal and local legislations on top of the professional code of deontology represented an excessive administrative burden. Most of the interviewees recognised that communication with external physicians, experts and politicians was difficult. One interviewee associated this communication with the word “preaching” in order to illustrate the effort required to convince others that the data are of good quality, representative, and provide real-life evidence, i.e. that people could trust the CRG results and apply them in their daily work. Group R considered the other HCSs as somewhat inept given their relatively poor legal and ethical knowledge, and a reluctance to share data.

The issue of data sharing and networking was regularly mentioned but difficult to clarify with interviewees: On the one hand, they showed a willingness to harmonize definitions and standardise electronic entries to ensure the quality of the CRG. On the other hand, they appeared reluctant to communicate with information technology specialists, who were considered insufficiently capable to understand and subsequently translate medical information into standardized items. Furthermore, some respondents recognized that transparency could be perceived as another obstacle to data sharing, because physicians may prefer non-transparency. Finally sharing CRG data was not synonymous with linking registries to create bigger data sets. The interviewees close to patients insisted on the importance of meaningful information. They sought to provide bottom-up inputs to data centre managers and steering committees in order to help make the findings understandable and meaningful. For them, “big” was not clinically interpretable and useful for their practice.

ii. Attitude towards patients’ role and agency (Table 5)

**Table 5:** Healthcare professionals’ attitude towards patients

Perception of patients’ capacity	Interviewee comments (A, M’, M”, R indicate group affiliation)
Patients want to know	[A] [translation] *“Patient information is the most important. Patients need information in order to be able to consent or refuse. Information should be in a language they could understand.”* [M”] *“We have asked the physicians and the patients what they thought about that, and they were saying: well I am not against but I want to be informed, I want to be informed preferably by my family physician, by my GP and I want to know what is going on, but I am not opponent to this type of research, but I would like to know.”*
Patient information is not systematic	[Sub-group M”] *“We have a formal agreement [from the federal commission for professional secrecy] that the data can be transmitted to us. They are anonymous, and the patients don’t know.”*
Not much value assigned to patient’s capacity to understand	[Sub-group M’] [translation] *“Well, for me it is important to inform patients …yes the patients, even if they usually don’t care about it.”* [A] [translation] [interviewer’s question about the perception of patient’s position] *“The patient? it is eight million of citizens, and each of them have their head, their morality, their feelings, their perception of the reality… There is no patient lambda …If I had to answer your question, I would say that a patient lambda in Switzerland has no idea about what you are asking- It is a level of mental abstraction that is present in less than 1% of the population.”*
Patient information is a moral duty	[Sub-group M’] *“We do not need [an informed consent] because we, basically we collect data which is collected anyway, so you could argue the patient doesn’t really care, but he needs to know what it’s done, you know.”*
Patient information is useful to maintain trust	[Sub-group M’] “*A well informed patient is convinced that he can really trust how his data is handled, about security of the data. He will be more willing to say yes; I agree that my data will be put into this database.”*
Patients have the capacity to contribute actively	[Sub-group M”] *“We also plan to have a, in a second line, a patient self-registry, so that the patients themselves can register themselves into the registry. So, there is two ways to go in. So, either for the doctor, physician, or then for the patient himself: I have this rare disease, I want to be part of this registry.”*

All interviewees recognized patient rights to know, to protect privacy and to own their data. However, their attitude regarding patient information indicated some discrepancies with this position. No clear answer was given to the question of the destiny of data following patient withdrawal. Also, the possible risk that patient information could be neglected in the absence of formal consent was accepted – for instance, when a data centre treated their data anonymously, patients were not supposed to be informed.

Despite our research design including the prompt cards “patient information” and “trust”, respondents didn’t attach much importance to empathy with patients, patient information or patient agency; many interviewees thought that patients did not care about or could not understand CRGs. Exceptions to this attitude concerned one participant who belonged to a patient association in the discriminative sampling, as well as most of the treating physicians who – in comparison to other HCSs – indicated greater concern for patient information in the interests of building and maintaining a trusting relationship with their patients. No respondents were ready to accept patient membership in steering, nor they think that patients could act as members of a governance body. They never mentioned the possibility or need to empower patients’ agency. Some interviewees remarked that patient associations were weak and that patient participation in a steering committee would only be an “alibi” and have no added value. A few respondents stated that patients were too “self-centred” to be able to participate. Only one interviewee spoke about the possibility of patients registering themselves in a CRG, in the special context of rare diseases.

iii. Attitude towards society (Table 6)

**Table 6:** Healthcare professionals’ attitude towards society

Perception of society benefit & role	Interviewee comments (A, M’, M”, R indicate group affiliation)
Patients are citizens	[A] “*The patient is all of us”.*
Clinical registries benefit society	[Sub-group M’] *“Primarily it’s a tool for everybody who has a research question “****.***
Patients may benefit directly	[Sub-group M”]: “*It is always known that patients in a registry, they are usually better followed than other patients. ...because we have to see them every half year [in the cohort].”*
Social value is related to meaningful use	[Sub-group M’]: *“You want to do that in a meaningful way; I mean we don’t do it in a sense that the DRG system; all the patients get a DRG kind of diagnosis, you know. So, in the end of the year you can bring all these DRG diagnosis together. But they are worth not that much. Obviously, they are worth very much because you are paid according to the DRG so it’s important, but in terms of what they really say, what the patient has, and as outcome, it’s, it’s, you can’t use it.”*
Value increases with data sharing	[R] *“Sharing data increases the value of the collected clinical registry. A good register communicates, publishes results and invites further research proposals on these data.”*
Value needs better physicians’ education	[R] *“Physicians should understand the value of what they do when they use epidemiological data. Most physicians feel the moral obligation to keep registries for good quality assurance, but they tend not to share them, not to be transparent. Therefore, comparison is not possible and quality could not be improved. We should have a better national medical education and training, including evidence based medicine, epidemiology and the practice of critical thinking and reflection. Continuous education as well for medical development.”*
Value includes financial risk-taking	[A] [translation] *“There is always a risk of error in the long term. I have some colleagues who told us that investments have to be made only in research projects that we are sure in advance that they would provide results. They have a serious problem understanding the word research.”*
Value is related to trust	[A] [translation] *“Here, the socialist party will say: we need a beautiful law that ensures financing, governance, and an interdisciplinary governance which controls everything…*etc. *and which costs three times more. What the right side says is: no, we provide a legal basis and let the people free, and if they make mistakes, there are enough means to address them …it is this vision that I called the principle of trust.”*
Value and governance	[Sub-group M’] *“The CRGs should have a medical and social value. The Federal Office of Public Health is not apt to do it. It could financially support CRGs but only professional societies could govern CRG.”*
Conflicts in interest	[A] *“Transparency issue is a possible deceptive motivation for a registry. CRG may be advocated for patient interests, but in fact would be performed and used for publications and academic careers of the investigators first of all.”*

A few interviewees explained that patients disliked the label “patient” as they were hoping for recovery to “normal”. They explained that patients would like to be part of the overall citizen community, i.e. the society. Respondents acknowledged CRGs’ societal value as the delivery of public benefits and the possibility of improving medical knowledge. They recognized that, depending on their purpose, all health stakeholders could benefit from CRGs. Physicians and hospitals would improve their work, researchers would identify new patterns, experts would make better-informed recommendations, public health players would justify their decisions and health regulations, and insurers might introduce premium rebates. As a result, patients in general would benefit from all of these improvements, i.e. patients’ family and future patients. Some interviewees mentioned that patients themselves might secure direct benefits as participants of longitudinal cohorts could benefit from better medical follow-up, as they are regularly contacted.

For most of the respondents, CRG data sharing and the dissemination of this information increase the social value of CRGs. The belief in these public benefits justified public funding and governance. Group R was particularly supportive of moral obligations concerning transparency, openness and comparative effectiveness between HCSs. To this end, it was suggested that physicians needed to be better trained in information technologies and public health sciences. Group A focused as a matter of priority on addressing two major risks: privacy infringement because of excessive transparency, and the waste of data because of data cemeteries and low cost-utility ratio. Perspectives of the waste of data were dependent on the political standpoint of policy-makers from group A. Those from the right and liberal political parties preferred to restrict public spending and emphasized the risk of waste of data. In contrast, the representatives from the political left were more open to take on a financial risk to develop long-term CRG research in order to measure and improve equity in access to care. It seems that conservative policymakers believe in public interest for patient data but do not equate this with public funding.

Governance was an important issue, but subject to conflicting views. Most interviewees supported public governance to serve public interest. For them, CRG resource allocation should remain scientifically and academically driven and be free from conflicts of interest, as in the case for projects supported by the Swiss National Research Fund. A few interviewees were in favour of small, non-bureaucratic governance, independent from institutional, economic or political powers, i.e. public authorities would provide financial support, but would not be involved in CRG governance. For group R, governance was not the role of an ethical committee, but it required a common long-term political vision including the prioritization of CRG projects, and an ethics of responsibility for each physician and patient.

The definition of “public” was restricted to institutions and public associations or academies. No interviewee considered representatives of civil society as potential members of governance organisations for CRGs. Nor did they support health data literacy programs to facilitate the participation of citizens, even when the question was directly asked. One interviewee said that the necessary education should be done in school and not later, because it would not be feasible or effective with patients or adult citizens. The role of communities and society was mainly limited to financing CRG via cantonal and federal contributions, and sometimes, unusual health contributors such as the national lottery. Moreover, lobbying or private funding were seen as risking unfair allocation of healthcare resources.

#### Respondents’ suggested strategy

When they came across moral conflicts between norms guiding their behaviour and their attitudes, interviewees started to develop practical strategies to support decision-making. All respondents wanted to improve the utility of the CRGs, i.e. applying norms to increase the number of included patients, stimulate physicians’ participation, improve data quality and interoperability, and align legal and ethical guidelines amongst CRG stakeholders. Nevertheless, when confronted with moral conflicts, there was no consensus on which specific strategies to adopt, as shown in the following examples:

- Strategies towards enhanced patient recruitment were dependent on HCSs’ beliefs on the balance between patient rights and public interest. Sub-group M’ relied on better patient communication to develop more trustful relationships in order to include more patients. Sub-group M” believed in their capacity to influence policy-makers to promote opt-out forms of consent and new forms of consent. A few suggested using financial strategies either as incentives (pull strategy) or as penalties (push strategy) for investigators.
- When confronted with the doubt about the distinction between care and research CRG, tensions were apparent and attitudes diverged. The majority chose the definition of a care CRG in order to avoid the administrative burden of a research ethical committee, this potentially at the expense of patient rights. Only a minority of HCSs implemented a default-strategy of systematic declaration to the research ethical committee. This latter attitude was also motivated by the desire to be protected from any legal risk due to infringement of the HRA.
- Tensions were present regarding data ownership. All respondents considered patients as data owners, but only a minority of HCSs thought that patients ought to have some kind of compensation rights for the use of their data. Moreover, contrasting concepts were envisaged to facilitate the use of data. Group M favoured stewardship towards their patients’ data, whereas group A preferred a split “puzzle” approach to data ownership avoiding power concentration.

#### Final analysis and emergence of an initial theory

**Fig. 1:**
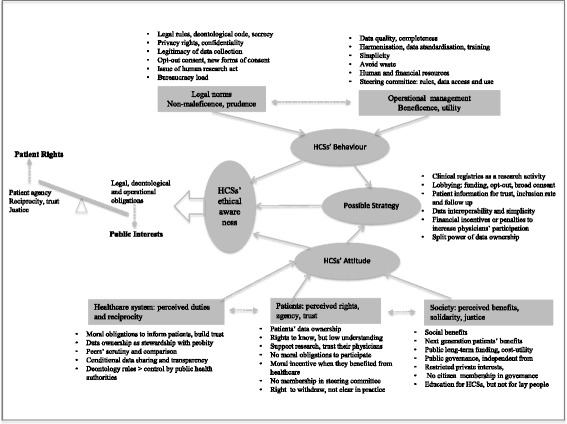
Thematic organisation for in-depth understanding

The reflection on the former themes has led to a better understanding of HCSs’ ethical awareness. Figure 1 organised the themes on one sheet of paper (OSOP) which served as the basis for reflection to search for patterns, links, comparisons or oppositions, and explanations with the aim of establishing the story in each theme and developing perspectives for an all-inclusive final interpretation.

The qualitative interpretation supports the passage from a thematic description to an initial theory regarding the research question. The proposed theory about HCS’s ethical awareness was based on the following three interpretative perspectives:

i. A perspective of professional needs and public interest. HCSs concentrate on legal norms, deontological code, operational data management rules and good clinical practice, in order to ensure the legitimacy and utility of the collection and use of patient data necessary for the different types of CRGs. Their ethical justification relies on the principles of non-maleficence and prudence as well as social utility and benefits. In contrast, they give little consideration for patient rights to be adequately informed, i.e. to be informed even when consent is not required, to receive feedback, to have the right to ask questions and the right “not to know”. HCSs argue that patients have no moral obligations to participate in CRGs, but at the same time they try to stimulate participation by calling for opt-out procedures and new forms of consent. HCSs’ ethical awareness seems to be more guided by their professional needs surrounding the collection and use of patient data than by an interest in the protection of patient’s rights and autonomy. From a normative point of view, this utilitarian perspective may impede an adequate assessment of the possibility of infringing patient rights for the sake of professional and public interests.
ii. A limited perspective on transparency and trust between HCSs and with patients. Communication between HCSs is problematic and compromises peers’ trustworthiness, because they do not support unconditional transparency and scrutiny of their work, and show limited interest in bottom-up inputs. Communication with patients raises a similar issue. HCSs do not assign much value to the patients’ capacity to understand the complexity of the production and use of large patient data sets, and therefore do not foster reciprocal trust with patients. These limitations with transparency and trust increase the risk that peers are reduced to simple data collectors, and patients to simple data sources in the production of a CRG. These difficulties with recognizing patients as fully capable of shared decision-making indicate that HCSs’ interests for patient data could overcome the consideration of patients as an end themselves. The potential utilitarian bias in favour of research and public interests noted above is therefore insufficiently balanced by a more Kantian deontological approach forbidding the treatment of individuals merely as the means to others’ ends.
iii. A perspective of governance by experts at the expense of citizen participation. HCSs have demonstrated little overall awareness of the role of citizens in the governance of CRG patient data and the need to develop citizens’ education for such a role. HCSs don’t foresee the involvement of civil society representatives in governance. They recognise the need for public funding, but not the possibility that society could contribute to assess the whole range of social benefits and risks, i.e. balancing privacy against public interests. From a normative perspective, the process of deliberation and decision-making thus remains unduly expert-centred, favouring research, economic and political objectives, with a risk of conflicts of interest, hidden agendas, and biased decisions regarding distributive justice.

The resulting initial theory states that HCS could be more aware of the potential tension between patient rights and public interest around patient data. It is summarily represented with the scales of public interest vs patient rights on the left of Fig. 1. This initial theory will be examined and enriched in the following section.

## Discussion

This exploratory qualitative research brought to light a potential tension between public interest and patient rights regarding patient data. Patients are solicited to give up rights to their health-related data, but their participation appears restricted to their role as data donors, with little recognition of the possibility of meaningful participation in decision-making. This potential tension carries the risk of weakening trust in the patient-physician relationship, undermining solidarity and justice at the societal level, and unfairly infringing upon patient rights. Therefore, the transferability of these findings to other debates regarding the use of patient data in the healthcare system needs to be examined.

No other qualitative study to date has explored the subject in Switzerland. However, our findings are in line with the current evolution of the Swiss healthcare system. First, the Federal Act for National Oncology Registry, approved in March 2016, will ease the production of patient registries with an opt-out procedure [15]. Second, operational recommendations for health-related registries have been published in July 2016 to ensure data protection and data quality, appropriate information and management, and cost/utility of CRG data (http://www.anq.ch/fileadmin/redaktion/deutsch/20160926_Empfehlungen_Register_final_en.pdf) .Finally, a broad informed consent for all secondary research usages of samples and related patient data has already been implemented in some public hospitals, and is on its way to be implemented nationally [16]. All these events were in discussion at the time of the interviews, and it is thus difficult to determine whether the events have influenced the interviewees or if the reverse is true. Nevertheless, there has not been a concomitant development of ethical considerations promoting patient agency and consumer participation in the management of patient data. Nor has the new national project of Swiss Health Personalized Network involved patients or donors in its governance [17]. Furthermore, a Swiss parliamentary motion requiring a legal approach to ensure patient representation in the governance of biological data has recently been raised but rejected, confirming the pertinence of our findings in the current Swiss context [18].

Previously published literature confirms that healthcare professionals and administrators give little attention to patients’ ability to understand or contribute to governance. The qualitative studies referred to in the design stage of our study interview guide were conducted in the UK and North America [19–29]. Similar to our findings, healthcare professionals and administrators in these studies were vigilant about data protection rules, data security, confidentiality, responsibilities for patient data, and they strived for simplicity in data processes, transparency, and consensual rules between peers. These studies also included focus groups or interviews with patients. Yet, patients’ opinions showed some divergence with other HCSs’ perceptions. Patients accepted the sharing of their data, but wanted to be informed and to have the freedom to participate or withdraw. They did not want their data passed to third parties, insurance or pharmaceutical companies, without being specifically asked beforehand. Patients usually favoured trust and partnership with their treating physicians. In fact, it seems that citizens in general, as well as patients in specific, have the capacity to intervene and assess the just equilibrium of patient rights and public interest concerning the use of personal health data. In Australia, a country with a long history of clinical registries, community and consumer representatives already participate in the governance of clinical registries [30]. More recently in the USA, following the Obama initiative on precision medicine, community citizens have participated in the registry design of the “one million Americans” cohort project [31]. Indeed, Fair Information Practices Principles (FIPPs) have been developed to set common standards for patient involvement in the governance of personal health and genomic data [32].

Assuming the transferability of our findings to all health-related big data, the tension between legal, professional and operating norms on the one side, and respect for patient agency and citizen contribution on the other side, could be difficult to resolve in the absence of additional ethical guidance. There is a risk that those with the expertise or economic power to effect change might favour additional legal and operational norms, to authorize the use of patient data for research and public interest. Thus, purported social benefits could justify ever-greater usage of patient data, at the expense of listening to patient voices. This could lead to an erosion of the necessary reciprocity, solidarity and trust in the healthcare system. Consequently, more data sharing, justified on the grounds of social benefits, could foster distrust towards healthcare professionals and the public healthcare system in general, thus reducing the expected social benefits.

The identification of this potentially negative scenario is stimulating new ethical approaches. The World Medical Association (WMA) has thus raised concerns about the management of large patient data sets and biobanks, some relating to privacy, and others relating to patient autonomy and dignity, and to commercial issues [33]. WMA recommends that the collection and usage of patient data should require patients to be properly informed, with a clearly defined set of information about how their data will be used. Further, WMA recommends that when this is impracticable, there must be a governance process that protects patient rights across all future uses of their data. And, a broad consent agreement should not be unconditional. These recommendations primarily concern physicians. Nevertheless, they could be broadened to other HCSs and integrate patient and citizen concerns. This integrative approach can already be spotted in the literature. Ethical, legal and social implications (ELSI) focus on topics of consent, disclosure, data sharing, privacy and confidentiality at a population level [34]. However, as a complement, in its 2015 report, the Nuffield Council on Bioethics recommended the usage of four essential principles - respect for the person, respect for established human rights, participation of those with morally relevant interests, and accounting for decisions – that integrate bottom-up ethical considerations to biological and health data [35].

Similar ethical reflections have already been developed when a tension has arisen between clinical research for public interest (framed with a utilitarian approach) and individual care (based on a deontological framework and patient rights). It was advocated that patients’ values and knowledge should also be considered when their data is used [36]. In the case of governance of big biobanks, Brownsword proposes to have recourse to a “mini-constitution” that simultaneously protects patients from disproportionate claims of public interest, whilst introducing greater flexibility for future research [37]. This human-rights-based approach, with greater patient involvement, could be applied to all large patient data sets. It is acknowledged that patients and scientific citizens should have an increasing role in the management of human genetic and health databases in a democratic society. Citizens’ participation would therefore require the development of public programs for health digital education, and public spaces for deliberation and citizen consultation [38].

Whilst it is beyond the scope of this paper to enter into a detailed normative development, we think that the approaches presented above, that emphasize the need to involve patients and citizens, provide us with a good starting-point for further ethical reflections with regard to the production and use of large patient data sets. Our paper is to be read neither in favour of nor against one specific group of stakeholders. Identifying a tension can be positive in cases of complex situations, helping to advance a change in perspective.

### Limitations and future steps

The final analysis of this study must be considered with caution, as empirical findings are not directly translatable into normative ethics. During the interviews, we could not avoid a gender effect, which reflects the actual Swiss situation of fewer women than men in leadership positions. Interviewees were also predominantly physicians, as often observed in the healthcare domain. These observations on gender and profession may explain the traditional and deontological perspectives of the Swiss HCSs regarding patient rights and patient passivity. We did not have access to the patients enrolled in the interviewees’ CRGs because of anonymity rules. Patients were thus not interviewed, with the exception of one patient organisation representative from the discriminative sample. As a next step, it would be paramount to investigate the patient perspective directly, and the evolution of the patient-physician relationship when confronted with the complexity of the creation of large patient data sets, as well as the use and the return of the resulting information. However, it is not always easy to study patients, as they can be too focused on specific pathologies or possibly under the influence of for-profit industrial lobbies [39]. Our findings, showing consequences for the whole of society, argue for broad representation from the community in future empirical investigations.

## Conclusion

The aim of this research was to gain an in-depth understanding of how those involved in the collection and use of patient data - healthcare professionals, regulators and policy makers - were aware of related ethical issues. Transferable research findings can be recapitulated as follows: First, a utilitarian inclination towards norms and guidelines aiming at facilitating the collection and use of patient data appears insufficiently balanced by a duty-oriented deontological approach to patient rights; second, HCSs did not assign much importance to patient rights in terms of patient agency and their ability to share steering decisions; third, at the societal level, it follows that there is a pre-eminence of experts’ role in the governance of large patient data sets at the expense of consumers’ representation. These findings raise ethical questions regarding patient rights, social justice and trust in public institutions that might undermine the expected social benefits of data sharing. For these reasons, HCSs should be more aware of the identified tension between public interest and patient rights, with further ethical guidance being required. Rather than setting patient rights against public interest, new ethical approaches would aim to strengthen both concurrently, advancing long-term sustainable cooperation.

## Additional files

Additional file 1: Definition of clinical registries for the purpose of the qualitative study. This data includes a definition of clinical registries based on the U.S. Agency for Healthcare Research and Quality (AHRQ) document, and a matrix summarizing the definition and taxonomy of clinical registries [6]. At the time of the interview, this definition was read and the interviewees were asked to fill in the matrix to identify their own CRG experience. (DOCX 104 kb)

Additional file 2: Literature research strategy for the qualitative research. This file illustrates the ad-hoc literature review which identified an initial framework of ethical potential issues to be raised by CRG stakeholders, and helped develop topic guides and prompt cards for the semi-structured interviews. (DOCX 86 kb)

Additional file 3: Topic guide example for the group “M” making the clinical registries. This data shows an example of the topic guide used for the interview of the group M participants. The topic guides for groups A and R had a first part slightly different to fit with their own working experience with clinical registries. They are not shown but available on request. (DOCX 110 kb)

## Abbreviations

CRG: Clinical registry
DRG: Disease-related groups
ELSI: Ethical, legal and social implications
FMH: Swiss medical Association
GP: General physician
HCS: Healthcare stakeholder
HIV: Human immunodeficiency virus
HRA: Human Research Act
NIH: National Institute of Medicine
UK: The United Kingdom
USA: The United States of America
