# Supplementary material for: Patient data and patient rights: Swiss healthcare stakeholders’ ethical awareness regarding large patient data sets – a qualitative study
Source: BMC Med Ethics. 2018 Mar 7;19:20. doi: 10.1186/s12910-018-0261-x (PMC5842517; doi:10.1186/s12910-018-0261-x)
Supplement: Supplementary file 1 — Definition of clinical registries for the purpose of the qualitative study. This data includes a definition of clinical registries based on the U.S. Agency for Healthcare Research and Quality (AHRQ) document, and a matrix summarizing the definition and taxonomy of clinical registries [6]. At the time of the interview, this definition was read and the interviewees were asked to fill in the matrix to identify their own CRG experience. (DOCX 104 kb) [file 12910_2018_261_MOESM1_ESM.docx]

**Additional file 1. Definition of clinical registries for the purpose of the qualitative study**

***Definition of clinical registries after the Agency for Healthcare Research and Quality****

*A clinical registry is an organized system that:*

*1. produces healthcare data using observational study methods to collect uniform data (clinical and other), outcome-focused, from a population of patients defined by a particular disease, condition or exposure,*

*2. used for one or more predetermined purposes i) clinical care (measurement and improvement of the quality of healthcare services and process, monitoring of safety and harm, comparison overtime), ii) scientific research (knowledge/natural history of the disease, effectiveness of a drug, device or intervention in real-life, off-label uses of therapeutic products, recording of safety and unexpected event), or iii) policy (cost-effectiveness evaluation, health technology assessment, comparison between institutions or physicians) ,*

*What makes the specificity of a clinical registry is its purpose according to how their populations are defined, for instance:*

*• Product registry: assess patients (all or samples of-) exposed to therapeutic products (drugs or devices)*

*• Health services registry: assess clinical quality and outcomes in patient groups having a common procedure*

*• Disease or event registries: assess populations with the same diagnosis*

***** Glicklich RE, Dreyer NA, eds. Registries for Evaluating Patient Outcomes: A User’s Guide. 2^nd^ ed. (Prepared by Outcome DEcIDE Center [Outcome Sciences, Inc. d/b/a Outcome] under Contract No. HHSA29020050035I TO3.) AHRQ Publication No. 10-EHC049. Rockville, MD: Agency for Healthcare Research and Quality. September 2010.

***Definition and taxonomy of clinical registries***

| **Type of clinical registry CRG** | | | | **Population definition** | | | | | | |
| --- | --- | --- | --- | --- | --- | --- | --- | --- | --- | --- |
|  |  |  |  | **Product registry** (patients exposed to therapeutic products: drugs or devices) | | **Health services registry** (patients with a common procedure: looking at clinical quality assessment and outcomes) | | **Disease or event registries** (population with the same diagnosis) | |  |
| **Predetermined purpose** | **Clinical care** | Quality measurement & improvement |  | |  | |  | |  |  |
|  |  | Monitoring safety and harm |  | |  | |  | |  |  |
|  |  | Comparison overtime |  | |  | |  | |  |  |
|  | **Scientific research** | Disease knowledge & natural history |  | |  | |  | |  |  |
|  |  | Effectiveness of a drug, device, or intervention in real life |  | |  | |  | |  |  |
|  |  | Off-label uses of therapeutic products |  | |  | |  | |  |  |
|  |  | Recording safety and unexpected events |  | |  | |  | |  |  |
|  | **Policy** | Cost-effectiveness evaluation |  | |  | |  | |  |  |
|  |  | Health technology assessment |  | |  | |  | |  |  |
|  |  | Comparison between institutions or physicians |  | |  | |  | |  |  |
