# Supplementary material for: Patient data and patient rights: Swiss healthcare stakeholders’ ethical awareness regarding large patient data sets – a qualitative study
Source: BMC Med Ethics. 2018 Mar 7;19:20. doi: 10.1186/s12910-018-0261-x (PMC5842517; doi:10.1186/s12910-018-0261-x)
Supplement: Supplementary file 2 — Literature research strategy for the qualitative research. This file illustrates the ad-hoc literature review which identified an initial framework of ethical potential issues to be raised by CRG stakeholders, and helped develop topic guides and prompt cards for the semi-structured interviews. (DOCX 86 kb) [file 12910_2018_261_MOESM2_ESM.docx]

**Additional file 2. Literature research strategy for the qualitative research**

***May 2014***

| A. Clinical registries | AND | B. Ethical Issues | AND | C. Qualitative Research |
| --- | --- | --- | --- | --- |
| *OR*  *Clinical, patient, disease-registries*  *Medical records*  *Electronic health records Medical record linkage* |  | *OR*  *Informed consent, anonymity, confidentiality, privacy, data sharing, ownership, conflict of interest, bioethical issues, clinical ethics, ethical review, ethics research, principle-based ethics,*  *Ethics, morality.* |  | *OR*  *Interview*  *Focus group*  *Grounded Theory*  *Mixed methods*  *Phenomenological analysis*  *Thematic analysis*  *Narrative analysis*  *Discourse analysis* |
| Pubmed 38 | | | | |
| Web of science 31 | | | | |
| After duplicates removed 39 | | | | |
| After abstract reading 13 | | | | |
| After full-text assessment for eligibility 11 | | | | |
| [Reference number in the manuscript]. Paper’s first author. Journal’s name, year of publication, volume, pages. | | | | |
| [19]. Stevenson F et al. *Family Practice* 2013; 30:227–232. [19] | | | | |
| [20]. Baird W et al. *J Med Ethics* 2009;35:92–96. | | | | |
| [21]. Korngut L et al. *BMC Medical Research Methodology* 2013, 13:135. | | | | |
| [22]. Baskaran V et al. *Informatics for Health and Social Care* 2013; 38(3): 196–210. | | | | |
| [23]. Caine K et al. *J Am Med Inform Assoc* 2013;20:7–15. | | | | |
| [24]. Maiorana A et al. *Implementation Science* 2012, 7:34. | | | | |
| [25]. Wright A et al. *BMC Medical Informatics and Decision Making* 2011, 11:36. | | | | |
| [26]. Walker J et al. *J Gen Intern Med* 2009, 24(6):727–32. | | | | |
| [27]. Jenkings K N et al. *Informatics in Primary Care* 2007;15:93–101. | | | | |
| [28]. Barrett G et al. *BMJ* 2006, 332: 1068-72. | | | | |
| [29]. Robling M R et al. *J Med Ethics* 2004;30:104–109. | | | | |
