# Supplementary material for: Patient data and patient rights: Swiss healthcare stakeholders’ ethical awareness regarding large patient data sets – a qualitative study
Source: BMC Med Ethics. 2018 Mar 7;19:20. doi: 10.1186/s12910-018-0261-x (PMC5842517; doi:10.1186/s12910-018-0261-x)
Supplement: Supplementary file 3 — Topic guide example for the group “M” making the clinical registries. This data shows an example of the topic guide used for the interview of the group M participants. The topic guides for groups A and R had a first part slightly different to fit with their own working experience with clinical registries. They are not shown but available on request. (DOCX 110 kb) [file 12910_2018_261_MOESM3_ESM.docx]

**Additional file 3:** **Topic guide example for the group “M” making the clinical registries**

***Place: Date: Code:***

1. **EXPERIENCE WITH CLINICAL REGISTRY (IES): Sub-group M**

- How would you describe your role for the registry?
- Can you explain what is the main purpose of your CRG?
- When did you last have to solve a problem regarding the registry?
- Can you tell me more about that?
- Is it a typical issue you have to face?
  1. If yes: frequency? Burden? Possible consequences? Suggested solution?
  2. If no: what would be a usual /typical issue? Could you explain it further? Possible burden? Consequences? Suggested solution?
- How close to your idea, are the other people involved in your CRG? If not closed, can you explain to me why?
- Can you tell me more about the management / governance of the CRG?
- Who do you think benefit the most from this CRG? Why?

1. **EXPLORING GENERAL ISSUES**

- Some physicians and public health responsible persons say that it is difficult to start or run a clinical registry. Can you tell me why they may think that?
- Do you share their point of view? Why?
- I have picked up some of the issues they reported about CRG on these cards: …**see cards**. Can you look at them, and think aloud…
  - What do you think about these different themes?
  - What do they mean for you? Why?
  - Are they all relevant?
  - Could you put them in a sort of order? Or in relation to each other??
  - Could you give me an example of a main issue?
  - Could you please tell me more about …
  - Could you explain why the issue on the last card is not so important for CRG?
- How will you define “relevant”… (In relation to what or to who?)
- Is there one or more issues not addressed by these cards?
- If yes, can you write them on these white cards? …
- How would you arrange the cards now? (*Emerging ethical framework*)
- What do you think patients would think?
- How do you feel now with these themes for CRG?

1. **POSSIBILITY OF EDITING RECOMMENDATIONS** (if short with time: bold only)

- How easy or difficult would be to address the main issues you mentioned?
- What would be the best way to do?
- **Would you recommend developing guidelines on what you advise to do?**
- How easy do you think would it be to implement such a set of recommendations?
- Can you explain me why?
- What will you advise to do about that?
- Would you recommend national ethical guidelines? Why?
- If yes, **how do you see the best governance for the implementation and revision of such ethical guidelines?**

1. **DEFINITION TABLE** *(beginning or end of the interview with demographic questions)*

Looking at the general definition of CRG given in the information sheet, I have made a table. Could we please tick all the boxes relevant to your registry. 🡪 table

1. **CONCLUSION**

- Thank you very much for your collaboration, I hope you enjoyed this interview,
- Looking back at it, is there anything you would like to add…
- Possibility to re-contact him/her by mail or telephone if something appears unclear at the analysis.
